# Relationship Between High Serum Levels of Follistatin with Impaired Physical Function, and Severe Disease Activity in Rheumatoid Arthritis

**DOI:** 10.3390/ijms26178232

**Published:** 2025-08-25

**Authors:** Fabiola Gonzalez-Ponce, Jorge Ivan Gamez-Nava, Heriberto Jacobo-Cuevas, Juan Manuel Ponce-Guarneros, Edgar Ricardo Valdivia-Tangarife, Cesar Arturo Nava-Valdivia, Norma Alejandra Rodriguez-Jimenez, Melissa Ramirez-Villafaña, Eli Efrain Gomez-Ramirez, Sergio Antonio Gonzalez-Vazquez, Aniel Jessica Leticia Brambila-Tapia, Eva Maria Olivas-Flores, Sylvia Totsuka-Sutto, Ernesto German Cardona-Muñoz, Laura Gonzalez-Lopez

**Affiliations:** 1 Programa de Doctorado en Farmacología, Instituto de Terapeutica Experimental y Clínica, Departamento de Fisiología, Centro Universitario de Ciencias de la Salud, Universidad de Guadalajara, Guadalajara 44340, Mexico; fabiola.gonzalez@academicos.udg.mx (F.G.-P.); ivangamezacademicoudg@gmail.com (J.I.G.-N.); juan.ponce4091@academicos.udg.mx (J.M.P.-G.); norma.rodriguezj@academicos.udg.mx (N.A.R.-J.); melissa.ramirez@academicos.udg.mx (M.R.-V.); dr.efrain.gomez@gmail.com (E.E.G.-R.); stotsuka@hotmail.com (S.T.-S.); cameg1@gmail.com (E.G.C.-M.); 2 Programa de Maestría en Salud Publica, Departamento de Salud Publica, Centro Universitario de Ciencias de la Salud, Universidad de Guadalajara, Guadalajara 44340, Mexico; 3 Programa de Postdoctorado en el Departamento de Psicología Básica, Centro Universitario de Ciencias de la Salud, Universidad de Guadalajara, Guadalajara 44340, Mexico; hjacobocuevas@gmail.com; 4 Unidad Medica Familiar 97, Instituto Mexicano del Seguro Social, Magdalena 46470, Mexico; 5 Departamento de Neurociencias, Centro Universitario de Ciencias de la Salud, Universidad de Guadalajara, Guadalajara 44340, Mexico; ricardovaldiviatangarife@outlook.com; 6 Departamento de Microbiología y Patología, Centro Universitario de Ciencias de la Salud, Universidad de Guadalajara, Guadalajara 44340, Mexico; cesar.navavaldi@academicos.udg.mx; 7 Aparatos y Sistemas II, Decanato de Ciencias de la Salud, Universidad Autónoma de Guadalajara, Zapopan 45129, Mexico; 8 Hospital General Regional 110, Instituto Mexicano del Seguro Social, Guadalajara 44716, Mexico; sergiogonvaz@yahoo.com.mx; 9 Departamento de Psicología Básica, Centro Universitario de Ciencias de la Salud (CUCS), Universidad de Guadalajara, Guadalajara 44340, Mexico; aniel.brambila@academicos.udg.mx; 10 Departamento de Anestesiología, Hospital de Especialidades, Centro Medico Nacional de Occidente, IMSS, Guadalajara 44340, Mexico; eolivasflores@gmail.com

**Keywords:** rheumatoid arthritis, follistatin, physical function, functional disability, inflammation

## Abstract

Rheumatoid arthritis (RA) is a highly prevalent chronic inflammatory rheumatic disorder leading to functional impairment and sequels. The search for new biomarkers helping in detecting RA subjects of high risk of functional disability is required. Studies showing high follistatin levels in RA have been described; however, none of them have placed focus on the role of follistatin as marker of deteriorated functionality. We aim to identify whether follistatin concentrations could be a potential biomarker of physical disability and disease activity in RA patients. Fifty-seven female RA subjects and 20 age–gender-matched controls were included in a cross-sectional evaluation. An assessment of clinical characteristics, grip strength, gait speed, and muscle mass was conducted. In RA subjects, disability was assessed using HAQ-DI and active disease using the DAS28-ESR. Follistatin levels were measured by ELISA. We compared (a) RA + functional disability and (b) RA + preserved physical function. Serum follistatin levels were increased in RA subjects compared to controls (175 ± 119 vs. 133 ± 47; *p* = 0.030). Follistatin levels correlated with deteriorated physical function levels (r = 0.491; *p* < 0.001) and severe activity (r = 0.344; *p* = 0.009). The RA + functional disability group, as compared to the RA + preserved physical function group, had higher serum follistatin levels (218 ± 159 vs. 141 ± 59; *p* = 0.030), lower grip strength (7.9 ± 4.6 vs. 14.5 ± 5.1; *p* < 0.001), reduced gait speed (0.77 ± 0.20 vs. 0.92 ± 0.20; *p* = 0.010), as well as higher proportions of tender joints ≥4 (48% vs. 16%; *p* = 0.008), and higher disease activity scores (3.8 ± 1.5 vs. 2.8 ± 1.2; *p* = 0.008). We concluded that higher follistatin levels are associated with physical functional impairment and the severity of disease activity in women with RA. Future studies are required to evaluate whether these follistatin levels can be related to other outcomes such as labor disability, hospitalization, and falls.

## 1. Introduction

Rheumatoid arthritis (RA) is a systemic chronic autoimmune disorder characterized by chronic inflammation of the synovial joints that can lead to progressive destruction of cartilage and subchondral bone, and extra-articular complications [1,2]. In a high number of patients with the disease, it can diminish patients’ physical functionality making it difficult to adequately perform daily tasks, such as writing, dressing, walking, or working, leading to falls and a high risk of hospitalizations, limiting consequently, their quality of life [3]. Impairment of physical function is a common feature of the disease observed in a third of patients with long-term or severe disease [4]. To determine the presence and severity of the abnormalities in physical function both clinicians and researchers use questionnaires and instruments. Of them, the most utilized is the Health Assessment Questionnaire Disability Index (HAQ-DI), which was developed to investigate the impact of RA on physical function. This instrument has been validated in several languages, including Spanish [5]. Using the HAQ-DI, it has been observed that an impairment in physical function in RA is around 56% [6]. Nevertheless, these instruments based on questionnaires depend entirely on the patients’ responses and these scores can be affected by other factors, including depression, fatigue, or other concomitant diseases that can modify their results. Therefore, the search for other objective measures related to disability is still ongoing and new biomarkers that can aid in the objective assessment of functional disability are required for RA [7].

Impairment in physical functioning in RA is related to abnormalities in skeletal muscle with loss of muscle mass an increase in fat infiltration of intramuscular fibers leading to muscle disfunction decreasing the capacities to perform daily life activities [8]. There are several recognized mechanisms of muscle damage in RA: (1) proinflammatory status with cytokines such as tumor necrosis factor (TNF)-α, interleukin (IL)-1β, and IL-6, that promote the degradation of muscle proteins and decreasing muscle regeneration; (2) the autoantibodies including rheumatoid factors and anti-citrullinated protein antibodies that can amplify the inflammation; (3) an increase in the production of reactive oxygen species with oxidative stress attacking lipids, proteins, and DNA with cellular damage; (4) abnormalities in the secretion of myostatin followed by muscle degradation; (5) autoreactive T-cells reacting against muscle antigens; and (6) accumulation of intermuscular adipose tissue (IMAT) also known myosteatosis. This last mechanism has been insufficiently studied and the molecules involved in RA associated with myosteatosis are still not well known [8].

Follistatin, a member of the Transforming Growth Factor (TGF) β family, is a glycosylated plasma protein that acts as a decoy under normal conditions, blocking the biological activity of activins and myostatin. The latter molecules have a role in the skeletal muscle degradation through binding to their activin type II receptors (ActRIIA/ActRIIB) on the cell surface [9,10,11]. Therefore, in normal concentrations follistatin is considered as protective, increasing muscle mass and muscle strength by promoting muscle repair and regeneration. Under physiological conditions, follistatin plays a pivotal role in skeletal muscle homeostasis by binding with high affinity to myostatin and preventing its interaction with ActRIIA/ActRIIB on muscle fibers [9]. This blockade disrupts SMAD2/3 phosphorylation, thereby averting the transcriptional repression of myogenic regulatory factors and inhibiting proteolytic pathways. As a result, protein synthesis is preserved, satellite cell activation is enhanced, and muscle fiber hypertrophy and regeneration are promoted. Normal follistatin levels are therefore closely associated with increased muscle mass and improved contractile performance [9].

However, under pathological conditions, particularly in elderly populations and patients with chronic kidney disease (CKD), high levels of follistatin can act negatively, being associated with inflammation and muscle wasting, increasing the risk of heart failure and mortality. Although follistatin is expressed in multiple tissues, hepatic production is predominant [12]. Once secreted, follistatin exerts systemic metabolic effects, such as reducing glucagon release and protecting pancreatic β-cells from apoptosis, ultimately increasing circulating insulin levels [12]. Its hepatic expression is stimulated by high glucagon-to-insulin ratios during fasting or exercise, and by FOXO1 activation under insulin resistance [13].

However, chronic hepatic overproduction can induce insulin resistance and trigger lipolysis in white adipose tissue, releasing free fatty acids that further impair insulin sensitivity [13]. This overproduction of follistatin can result in an infiltration of lipids into skeletal muscle tissue inducing accumulation and myosteatosis [14]. Myosteatosis is not only associated with metabolic disorders like insulin resistance, type 2 diabetes, and increased risk of cardiovascular disease, but also the abnormal accumulation of fat within skeletal muscle tissue, damaging muscle quality and reducing muscle strength and function, and negatively impacting mobility and overall function [14].

**Figure 1:**
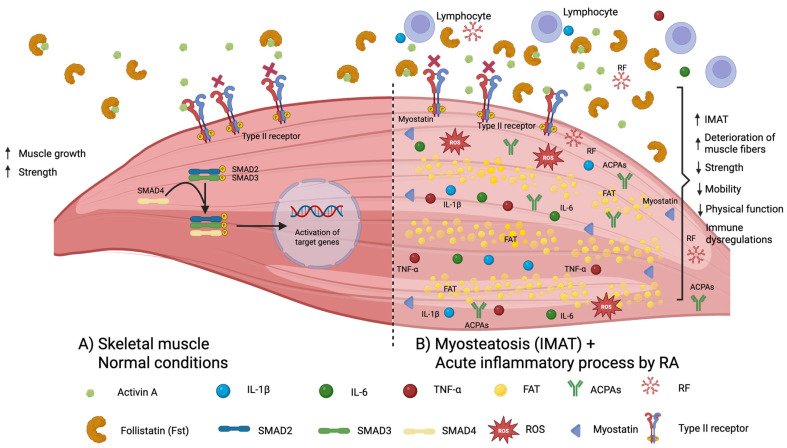
**Follistatin and other molecules in the skeletal muscle in normal conditions and in myosteatosis:** Abbreviations in the figure: IMAT: intermuscular adipose tissue; ACPAs: anti-citrullinated protein antibodies; RF: rheumatoid factor; IL: interleukin; TNF: tumor necrosis factor; ROS: reactive oxygen species.

Additionally, the interaction of follistatin with the main pro-inflammatory cytokines in RA, including TNF-α, IL-1β, and IL-6 (released by both fat cells and muscle cells), may promote a chronic inflammatory state within skeletal muscle, exacerbating intermuscular fat infiltration, impairing muscle function, and contributing to muscle atrophy and weakness [15,16]. Figure 1 shows the role of follistatin and other molecules in the skeletal muscle under normal conditions (A) and exemplifies the mechanism of muscle wasting and myosteatosis (IMAT) with the participation of excessive concentrations of follistatin and other pro-inflammatory molecules in RA (B). Of relevance for this study is the potential mechanisms of a hyperproduction of follistatin in a micro-environment with increased IL-6, TNF-α, and IL-1β. This leads to an intramuscular lipid accumulation with altered skeletal muscle metabolism, followed by deteriorations of muscle fiber, decrement of muscle strength, contractibility and mobility, and deteriorated physical function [17].

Studies performed in elderly populations without rheumatic disorders [18,19,20,21] and in patients with CKD, ref. [22], have identified that high levels of follistatin are associated with deteriorated muscle strength and impaired physical function [18,19,20,21,22]. High follistatin levels have been linked to frailty, decreased physical mobility, and poor exercise tolerance [18,19,20,23,24]. However, to date, there is a lack of studies regarding the potential role of follistatin in patients with rheumatoid arthritis and the limited information there is assesses a small number of number of patients. In a cross-sectional study focusing on Austrian patients, Kerschan-Schindl et al. found that RA patients (n = 24) had higher levels of follistatin compared to healthy controls (n = 24) [25]. In their study, high levels of follistatin in RA patients correlated with impairments in physical function [25]. However, their exploratory study provided no further information regarding the differences in follistatin levels between those patients with deteriorated physical function and patients with normal function and did not include a comprehensive assessment of other established measures of muscle wasting and physical impairment, such as grip strength or gait speed.

Therefore, the aim of our study was to evaluate whether follistatin levels could be a potential biomarker of physical disability, other measures of muscle wasting, and severity of disease activity in patients with RA.

## 2. Results

Table 1 presents the characteristics of female patients with RA (n = 57) and female control subjects (n = 20). A comparison between the groups shows that the RA group had a frequency of decreased grip strength (79% vs. 20%; *p* < 0.001), a greater decrement in gait speed (68% vs. 35%; *p* = 0.009), and lower physical performance (7.5 vs. 5.9; *p* < 0.001). In addition, serum follistatin levels were higher in the RA group than in controls (175 ± 119 vs. 133 ± 47; *p* = 0.030). The other assessed variables, such as age, menopause, body mass index (BMI), waist circumference, waist-to-hip ratio, skeletal muscle mass index (SMMI), and comorbidities, were similar for the RA group and the control group (Table 1).

**Figure 2:**
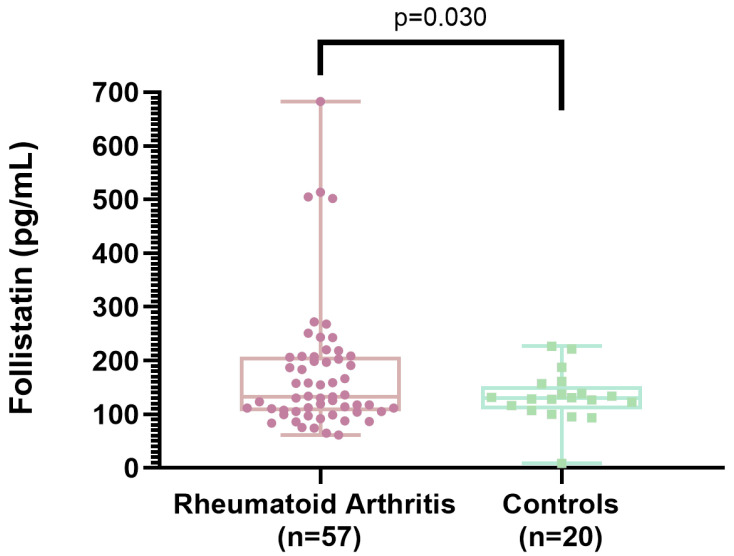
Comparison of serum concentrations of follistatin between rheumatoid arthritis patients and controls. The box and whisker plot shows levels on the Y-axis and the analyzed groups (RA vs. controls) on the X-axis. The central line within each box represents the median follistatin level for each group. The upper and lower edges of the box correspond to the upper and lower quartiles, respectively. The whiskers indicate the extreme values (minimum and maximum) observed for follistatin in each group.

Figure 2 shows the comparison of serum concentrations of follistatin between rheumatoid arthritis patients and controls. RA patients had a significantly higher follistatin concentrations (*p* = 0.030).

Figure 2 shows the means and standard deviations of serum follistatin levels in patients with RA compared to those in the control group.

Table 2 shows the results of two correlation analyses: (a) the first between serum follistatin levels and clinical variables in RA patients, and (b) the second between HAQ-DI scores, clinical variables, and follistatin levels in RA patients. Higher follistatin levels were positively correlated with lower physical function (HAQ-DI scores) (r = 0.491; *p* < 0.001), higher disease activity (DAS28-ESR) values (r = 0.344; *p* = 0.009), and elevated ESR levels (r = 0.398; *p* = 0.002).

In the second correlation analyses lower physical function (HAQ-DI) scores were positively correlated with greater waist circumference (r = 0.338; *p* = 0.011), deteriorated grip strength (r = −0.546; *p* < 0.001), limited gait speed (r= −0.307; *p* = 0.020), longer time to complete the physical performance test (r = 0.285; *p* = 0.032), higher numbers of tender joints (r = 0.356; *p* = 0.007) and swollen joints (r = 0.302; *p* = 0.022), higher disease activity (DAS28-ESR) values (r = 0.417; *p* < 0.001), higher ESR levels (r = 0.318; *p* = 0.016), and higher serum follistatin levels (r = 0.491; *p* < 0.001) (Table 2).

Table 3 presents a comparison of clinical characteristics between RA patients with functional disability and those with preserved physical function. This comparison shows that the group with functional disability had greater waist circumferences (94.5 ± 12.8 vs. 87.1 ± 11.6; *p* = 0.029), higher waist-to-hip ratios (0.92 ± 0.06 vs. 0.86 ± 0.08; *p* = 0.006), lower grip strength (7.9 ± 4.6 vs. 14.5 ± 5.1; *p* < 0.001), reduced gait speed (0.77 ± 0.20 vs. 0.92 ± 0.20; *p* = 0.010), longer durations to complete the physical performance test (8.3 ± 2.4 vs. 6.9 ± 1.8; *p* = 0.014), a higher proportion with a tender joints count of ≥4 (48% vs. 16%; *p* = 0.008), higher DAS28-ESR values (3.8 ± 1.5 vs. 2.8 ± 1.2; *p* = 0.008), and higher serum follistatin levels (218 ± 159 vs. 141 ± 59; *p* = 0.030). Regarding pharmacological treatment, the group with functional disability more frequently used prednisone at doses below 10 mg/day (64% vs. 35%; *p* = 0.025). The use of other pharmacological treatments did not differ between the groups (data not shown in the table). The other assessed variables—such as age, smoking, menopause, BMI, SMMI, comorbidities, RA disease duration, and the number of tender or swollen joints—showed no differences between the RA group with functional disability and the RA group with preserved physical function (Table 3).

**Figure 3:**
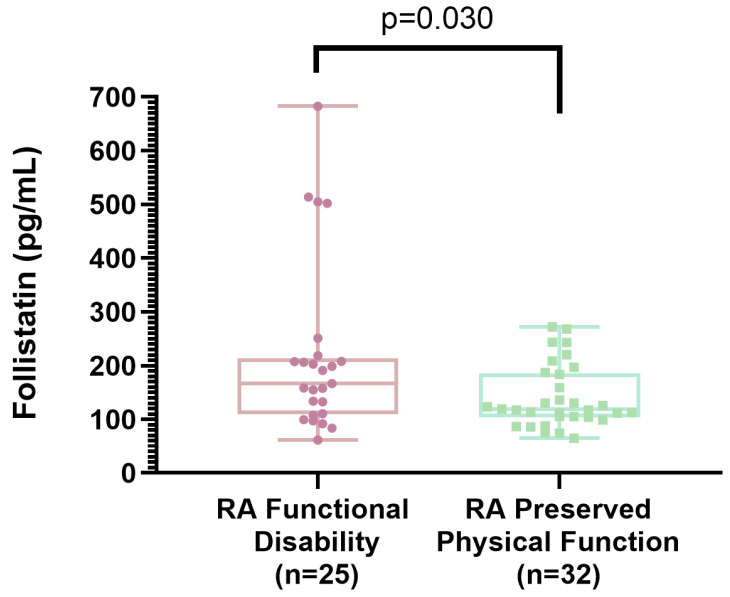
Comparison of follistatin levels between (1) RA + functional disability (defined by a HAQ-DI ≥ 0.6) and (2) RA + preserved physical function (defined by a HAQ-DI < 0.6). The box and whisker plot displays levels on the Y-axis and the analyzed groups (RA + functional disability vs. RA + preserved physical function) on the X-axis. The central line within each box represents the median follistatin level for each group. The upper and lower edges of the box correspond to the upper and lower quartiles, respectively. The whiskers indicate the extreme values (minimum and maximum) observed for follistatin in each group.

Figure 3 presents the means and standard deviations of serum follistatin levels in the groups with RA and functional disability versus those with RA and preserved physical function.

Table 4 shows a subanalysis comparing follistatin concentrations between users versus non-users of different drugs. As it was observed in this table, there were no differences in follistatin levels in those users of methotrexate, sulfasalazine, leflunomide, chloroquine, anti-TNF agents, and prednisone compared with non-users of these drugs.

Additional findings not shown in tables: There were trends toward higher serum follistatin levels in patients with: (a) ≥4 tender joints compared with <4 tender joints (218 ± 154 vs. 156 ± 98; *p* = 0.074) and those with (b) ≥4 swollen joints compared with <4 swollen joints (238 ± 166 vs. 161 ± 104; *p* = 0.063) (data are not provided in the tables).

## 3. Discussion

### 3.1. Follistatin Levels Between Rheumatoid Arthritis Patients and Controls

In the present study, functional disability affected almost the half of the RA patients (44%). In the comparison of RA patients versus controls, RA patients showed differences in the impairments in handgrip strength and gait speed, even though the amount of skeletal muscle mass was similar for the two groups. Interestingly, the serum levels of follistatin were higher in the patients with RA as compared to controls. These elevations in follistatin in RA have also been observed by Kerschan-Schindl et al., who evaluated 24 Austrian RA subjects in remission versus 24 healthy controls [25].

### 3.2. Correlation of Serum Follistatin Levels with Clinical Variables

We identified a correlation between serum levels of follistatin and the HAQ-DI score, the DAS28-ESR, and ESR levels. Similarly to our findings, Kerschan-Schindl et al. found a correlation between the HAQ-DI scores and serum follistatin levels. However, these authors did not examine whether follistatin was related to the DAS28 score or ESR level [25]. Despite the correlation observed between follistatin and HAQ-DI, follistatin had no correlation with gait speed, handgrip strength, or muscle function. These latter findings had no previous reports in RA patients and are different to those observed in elderly non-rheumatic subjects by Fife et al. and Liaw et al., [19,20]. These studies found in non-rheumatic patients had a negative correlation between follistatin levels, gait speed, handgrip strength, and muscle function.

### 3.3. Correlation of HAQ-DI Score with Clinical Variables

The widely used HAQ-DI is a standardized index used for quantifying the level of disability in rheumatic diseases. It is based on a self-administered questionnaire covering eight domains related to daily living activities. HAQ-DI scores vary from 0 (“no difficulty”) to 3 (“unable to do”). Higher scores indicate worse physical function [26]. To date, no previous works have made a comparison between RA patients with deteriorated function versus RA with normal function. In concordance with our hypothesis, physical impairment is associated with elevated serum follistatin levels.

De Sordi et al. conducted a study involving 50 Brazilian patients with dermatomyositis and polymyositis [27]. These authors reported that serum follistatin levels correlated with HAQ-DI scores [27].

Additionally, it was observed by us that HAQ-DI scores were correlated with greater waist circumferences and waist-to-hip ratios. Samaan et al. evaluated 116 Egyptian patients with knee osteoarthritis identified a correlation between their HAQ-DI scores and waist circumferences [28].

In our study, we found no significant correlation between skeletal muscle mass measured by Dual-energy X-ray Absorptiometry (DXA) and HAQ-DI scores. In contrast, Son et al. conducted a study in 335 Korean RA patients and identified a negative correlation between HAQ-DI scores and skeletal muscle mass using electric bioimpedance [29]. Melikoğlu et al. in a cohort of 40 Turkish women with RA described a negative correlation between SMMI values by DXA and HAQ-DI scores [30].

Regarding physical performance, the HAQ-DI score was associated with reduced grip strength, slower gait speed, and longer time to complete a 6 m walk. Similarly, Lopes et al. found a negative correlation of HAQ-DI score with grip strength in a sample of 28 patients with systemic sclerosis [31]. Whereas, Özsoy et al., in Turkish patients with RA found a negative correlation between HAQ-DI scores and gait speed [32].

Findings in our patients show that impaired functioning was associated with higher DAS28-ESR values and more tender joints. Hammer et al. reported significant correlations between HAQ-DI scores and both the number of tender and swollen joints [33]. Similarly, Feng et al., in RA with active disease, found significant correlations between HAQ-DI scores, DAS28 scores, and ESR levels [34].

### 3.4. Association Between Functional Disability in Rheumatoid Arthritis and Clinical Variables

When we categorized our participants into RA patients with functional disability and those with preserved physical function, follistatin levels were higher in RA with functional disability. Although no works have directly assessed the relationship between functional disability in RA and serum follistatin levels, several studies have explored this relationship in non-rheumatic older adult populations [18,19,20,21]. Fife et al., in a study of 56 women and 45 men over the age of 60 in Poland, identified a negative correlation of follistatin and measurements of strength measurements and time for completing the Timed Up and Go test [19]. Similarly, Liaw et al. identified that higher follistatin levels were related to slow gait speed in a cohort of 205 older adults aged ≥65 years in Taiwan [20]. By contrast, in a 6-week clinical trial involving 30 older Iranian adults, Pazokian et al. implemented functional training three times per week and observed an increase in serum follistatin levels accompanied by improved grip strength [21].

Under normal conditions, the biological role of follistatin is as a binding protein that neutralizes activins such as activin A and myostatin. This prevents their interaction with ActRIIA/ActRIIB and subsequent activation of intracellular signaling pathways, including the SMAD pathway, the Akt-mTOR-S6K axis, and inhibition of FOXO1/3 transcription factors, all of which converge on muscle catabolism [35,36]. Because it blocks these pathways, follistatin is generally associated with increased muscle mass and strength [37].

However, under pathological conditions such as aging or CKD, high levels of follistatin may shift the muscle fiber composition toward a predominance of fast-twitch type II fibers reducing the quantity of type I fibers associated with endurance, leading to impaired motor function characterized by decreased gait speed and deteriorated mobility and working capacity [19,38].

Skeletal muscle tissue is composed of two myosin heavy-chain (MyHC) isoforms: MyHC type I (slow-twitch fibers), which have a small diameter, and MyHC type II (fast-twitch fibers), which exhibit greater power output, higher contraction velocity, and a larger diameter than type I fibers [39]. Growth of type II fibers is induced by several stimuli include mechanical loading, whereas myostatin drives a transition from fast (type II) to slow (type I) fibers. In this context, follistatin binds to myostatin, neutralizing its activity and preventing this type II-to-type I fiber conversion. Nevertheless, type II fibers display limited antioxidant defenses and can therefore generate substantial amounts of reactive oxygen species [39]. Reactive oxygen species can injure myocytes, altering their structure and function, and promote lipid peroxidation of fatty acids infiltrating the muscle, thereby leading to loss of strength and muscle function [40].

Overproduction of follistatin results in myosteatosis decreasing muscle strength and physical function [14]. The interaction of follistatin with other proinflammatory molecules in RA can exacerbate the fat infiltration, accelerating the muscle atrophy and compromising the muscle function, causing atrophy and weakness [15,16].

In relation to physical performance and the HAQ-DI, we found that functional disability was associated with lower grip strength and slower gait speed. Thyberg et al. and Escalante et al., in two separate works, identified that functional disability was related to reduced grip strength and walking speed. Thyberg et al. evaluated 217 RA subjects, while Escalante et al. assessed 681 RA patients; both studies observed correlations between the HAQ-DI, grip strength, and walking speed [41,42].

Regarding clinical characteristics in RA, functional disability was related to ≥4 tender joints, elevated DAS28-ESR values, and a greater frequency of active disease. Similar findings were reported by Meng et al., who studied 547 Canadian subjects suffering early RA (<12 months) and active disease observing that the tender and swollen joints were associated with greater functional impairment and working deteriorated capacities [43]. In contrast, Krause et al., in a cohort of 99 American RA patients classified according to their HAQ-DI scores (≥1.04 vs. <1.04), found no significant differences the number of swelling or tender joints [44].

Finally, we observed that patients with functional disability had a higher frequency of prednisone utilization (≤10 mg/day). Pfeiffer et al. evaluated 1,733 RA patients receiving prednisone (mean: 5 mg/day) over nine months, reporting improvements in functional disability, evidenced by a 0.35-point reduction in HAQ-DI scores [45]. On the other hand, Krause et al., in a follow study of 2.5 years, performed on 99 American RA subjects (mean age 60 years, 77% women), with functional disability (HAQ-DI of ≥1.04) found that current or recent glucocorticoid use was related to higher risk of impairment in functioning [44].

### 3.5. Strengths of the Present Study

We present the main strengths of our study: firstly, we identified that serum follistatin levels were statistically different between RA and controls, and moreover between RA with functional disability and those with preserved physical function; secondly, we detected differences in grip strength and gait speed, as well as differences in low grip strength and slow gait speed, both between RA and controls. Moreover, these differences were high between RA + functional disability compared to RA + preserved physical function; thirdly, our study identified several variables correlating with serum follistatin levels and HAQ-DI; finally, we reported the anthropometric, functional, and clinical characteristics of RA + functional disability compared to RA + preserved physical function, using an HAQ-DI cutoff value of ≥0.6.

### 3.6. Study Limitations

The first limitation is our cross-sectional design, which did not measure changes in follistatin levels during disease progression. The second limitation is the lack of data on other inflammatory molecules, which limited our capacities to evaluate other components of inflammation possibly related to follistatin levels in our population. Third, our sample size is relatively small. Additionally, our study included only female participants, preventing us from assessing how serum follistatin levels behave in male RA patients with functional disability. Therefore, future studies are needed to better understand the behavior of follistatin in this disease. We encourage longitudinal studies evaluating follistatin levels throughout the disease course to investigate whether baseline levels can predict future impairment in functional capacity among patients with RA. Multicenter studies including male patients and individuals with early-stage RA are required to enhance the external validity of our findings. Furthermore, we propose future research to examine the interactions between follistatin, pro-inflammatory cytokines, and activin A, in order to elucidate the contribution of these molecules to impaired physical function in this patient population.

## 4. Materials and Methods

### 4.1. Type of Study

Comparative cross-sectional design.

### 4.2. Study Population

The inclusion criteria were as follows: (a) females, (b) >18 years of age, (c) diagnosis of rheumatoid arthritis according to the 2010 American College of Rheumatology (ACR) criteria [1], and (d) be able to perform the tests included in the study.

The exclusion criteria were as follows: (a) overlapping syndromes (RA + other connective diseases), (b) inflammatory myopathies, (c) cancer, (d) active infections, (e) hypothyroidism, (f) pregnancy or breastfeeding, (g) receiving prednisone >10 mg/day (or equivalent), (h) serum creatinine levels >1.5 mg/dL, and (i) serum concentrations >2-fold above the reference values of aminotransferases (ALT and AST). Additionally, patients who were unable to perform the functional assessments were excluded.

In this study, 66 patients were screened. Nine of them were excluded for the following reasons: four patients were unable to complete the history regarding RA disease evolution; two patients had a history of osteoporotic radius fractures, which prevented the assessment of grip strength; and for three patients, blood samples were inadequate for a quantification of serum follistatin. Therefore, 57 females with RA were accepted to participate. They were assessed in the Institute of Experimental and Clinical Therapeutics (INTEC) of the clinical setting Centro Universitario de Ciencias de la Salud, Universidad de Guadalajara.

### 4.3. Assessments of RA Patients

Patients were asked to answer a structured clinical chart that included their non-pathological personal history (e.g., physical activity, smoking), comorbidities, history of RA disease, and pharmacological treatment; undergo anthropometric measurements (weight, height, circumferences) and a physical examination (swollen joints, tender joints, physical performance, etc.); and answer questionnaires to assess their RA disease activity and the presence of functional disability.

#### 4.3.1. Assessment of Functional Disability

For the evaluation of functional disability, we used the Health Assessment Questionnaire Disability Index (HAQ-DI) [5]. HAQ-DI is a self-administered questionnaire consisting of 20 items classified in 8 categories, assessing the physical ability for performing daily activities [46]. Each item is scored on a scale from 0 to 3, where 0 indicates no difficulty and 3 indicates an inability to perform the activity. The scores for each category are averaged to obtain the final HAQ-DI score [46]. The patients were classified into two groups (1) RA + functional disability defined as a HAQ-DI ≥ 0.6, and (2) RA + preserved physical function identified by a HAQ-DI < 0.6 [5].

#### 4.3.2. Evaluation for Active Disease

This was evaluated using the 28-joint disease activity score erythrocyte sedimentation rate (DAS28-ESR). The score is calculated based on the assessments and counting of 28 joints for (a) tenderness and (b) swelling, (c) a patient global health assessment (0 to 100 mm), and (d) erythrocyte sedimentation rate (ESR) in mm/Hr [47]. These four variables are then entered into a formula that yields a score ranging from 0 to 9.4 [48]. Disease activity is considered to be in remission if the score is <2.6, low if ≤3.2, moderate if >3.2 and ≤5.1, and high if >5.1 [48]. A cutoff of 4 or more swollen joints is often used to indicate active disease [49].

### 4.4. Inclusion Control Subjects

Twenty consecutive subjects without rheumatic diseases matched by age and gender were included in the control group. People with diabetes mellitus and/or hypertension that were controlled and stable under medication were allowed to be included. Exclusion criteria were similar to those used in RA patients: (a) cancer, (b) active infections, (c) hypothyroidism, (d) pregnancy or breastfeeding, (e) creatinine levels > 1.5 mg/dL, and (f) ALT or AST concentrations > 2-fold above the normal. Additionally, those patients who were unable to perform the functional tests (grip strength and gait speed) were excluded.

### 4.5. Clinical Evaluation for RA and Controls

RA patients and controls without rheumatic disease were assessed for weight and height, BMI using the Quetelet formula [50], waist and hip circumferences, and the waist-to-hip ratio. Elevated waist circumference is defined as > 88 cm [51]. The appendicular lean mass of extremities assessed using the General Electric Lunar iDXA software version 16 (Madison, WI, USA). All measurements were performed following guidelines of the International Society of Clinical Densitometry [52]. Based on these data, the SMMI was computed by dividing the sum of the arm and leg lean mass values by the height in meters squared [53]. The result was expressed in kg/m^2^. For defining low SMMI a value of <5.4 kg/m^2^ was used in females [54].

Subsequently, handgrip strength and gait speed tests were used to assess physical performance:

#### 4.5.1. Grip Strength

Grip strength was measured using a Jamar-type hydraulic dynamometer (Model 5030J1, Sammons Preston, Bolingbrook, IL, USA) [55]. During the assessment, the patient was seated in a relaxed posture and the elbow flexed 90° with the wrist in a neutral position. The patient performed three maximal grip attempts with each hand, resting 60 s between trials. Among the three attempts the highest result was recorded [55]. Low grip strength was defined as ≤16 kg; this cutoff is related to difficulty performing daily activities [56].

#### 4.5.2. Physical Performance

To assess the physical performance of the patients, gait speed was used. The gait speed test consists of assessing the speed at which an individual can walk a predetermined distance, which has been standardized to 6 m. This test is used as a measure of functional mobility and as a predictor of fall risk [57]. To perform the test, the time it takes the individual to walk the 6 m distance, at either their usual or maximal pace, is recorded with a stopwatch. The gait speed is then calculated in meters per second dividing 6 m by the time taken to walk that distance. Less than 1.0 m/s is considered the cutoff point for identifying reduced gait speed [57].

### 4.6. Serum Follistatin Determination

Venous blood was collected by venipuncture in non-fasting participants. Blood samples were centrifuged at 3500 rpm for 15 min to obtain the serum; thereafter, it was stored at –80 °C until analysis. The serum follistatin levels were determined by the enzyme-linked immunosorbent assay (ELISA) technique using a human follistatin ELISA kit (MyBioSource, Vancouver, British Columbia, Canada; catalog number MBS9501843). This assay had a sensitivity of 23–3000 pg/mL.

### 4.7. Statistical Analysis

Sample size calculation was obtained to identify differences between RA subjects with functional disability compared to RA with preserved physical function. Using data obtained in studies of follistatin levels in patients with sarcopenia from our center, a minimum difference of 90 pg/mL in follistatin levels was assumed between RA patients with functional disability and those with preserved physical function, with an estimated standard deviation of 100 pg/mL. A power of 80% and a *p* value of 0.05 were set. Based on these parameters, the calculation determined that a minimum of 20 patients in the group with RA + impaired function and 20 in the group with preserved physical function was required to reject the null hypothesis. Quantitative variables are shown as means ± standard deviations (SDs), while qualitative variables are presented as frequencies (percentages). A Pearson correlation analysis was used for numerical variables and serum follistatin levels, as well as HAQ-DI scores, and the results are reported as “r” values. Chi-square tests were selected to identify significance in percentages between groups, and Student’s *t*-test was applied to assess differences in means between the two groups. Statistical analyses were performed using the SPSS software, version 24. A *p*-value of ≤0.05 was considered statistically significant.

### 4.8. Ethical Approval

This study was approved by Research Ethics Committee, Institute of Experimental and Clinical Therapeutics of CUCS, University of Guadalajara with a registration number CEI/482/2019, and it adhered to the standards established in the Declaration of Helsinki, Finland. All the patients provided informed consent voluntary.

## 5. Conclusions

Elevated follistatin levels are related to deteriorated function and active disease in RA. It is of relevance to investigate the role of follistatin in clinically important outcomes related to functional disability, such as falls, hospitalizations, and work-related disability. However, follistatin levels were not associated with the type of treatment; therefore, follistatin concentration cannot be taken as a guidance to select the Disease-Modifying Anti-Rheumatic Drugs (DMARDs) or anti-TNF agents in RA. Further investigations are required to evaluate whether modifications on follistatin levels fluctuate over the course of the disease and with functional status, as well as in response to treatment. Additionally, it is necessary to assess whether follistatin is associated with other cytokines related to chronic inflammation in RA, such as TNF-α and IL-6.

## Figures and Tables

**Table 1:** Comparisons in clinical variables and follistatin concentrations between rheumatoid arthritis subjects and controls.

Variables	RA (n = 57)	Controls (n = 20)	*p*-Value
Age, mean ± SD	57 ± 8	56.5 ± 8	0.781
Menopause, frequency (%)	43 (77)	15 (75)	1.000
Diabetes mellitus 2, frequency (%)	7 (12)	2 (10)	1.000
Hypertension, frequency (%)	20 (35)	4 (20)	0.210
Body mass index (kg/m^2^), mean ± SD	27.3 ± 4.9	27.0 ± 4.5	0.788
Waist circumference, mean ± SD	90.2 ± 12.6	89.3 ± 9.5	0.783
Waist-to-hip ratio, mean ± SD	0.89 ± 0.07	0.86 ± 0.06	0.274
Skeletal muscle mass index (Kg/m^2^)	6.1 ± 1.0	6.2 ± 1.1	0.485
Grip strength, mean ± SD	11.6 ± 5.9	22.2 ± 8.0	**<0.001**
Decreased grip strength ≤ 16 kg, frequency (%)	45 (79)	4 (20)	**<0.001**
Gait speed, mean ± SD	0.86 ± 0.21	1.03 ± 0.16	**0.001**
Decreased gait speed < 1.0 m/s, frequency (%)	39 (68)	7 (35)	**0.009**
Physical performance, mean ± SD	7.5 ± 2.2	5.9 ± 0.95	**<0.001**
Functional disability (HAQ-DI ≥ 0.6) frequency (%)	25 (44)	-----	------
RA disease activity (DAS28 > 2.6), frequency (%)	27 (47)	-----	------
*Treatments:*			
Methotrexate, frequency (%)	52 (92)	-----	-----
Sulfasalazine, frequency (%)	41 (72)	-----	-----
Leflunomide, frequency (%)	29 (51)	-----	-----
Chloroquine, frequency (%)	35 (61)	-----	-----
Anti-TNF agents *, frequency (%)	19 (33)	-----	-----
Prednisone ≤ 10 mg/day, frequency (%)	27 (47)		
Follistatin (pg/mL), mean ± SD	175 ± 119	133 ± 47	**0.030**

Abbreviations: HAQ-DI: Health Assessment Questionnaire—Disability Index; DAS28-ESR: disease activity score of 28 joints—ESR. * Anti-TNF agents: etanercept n = 11 (19%), abatacept n = 1 (2%), adalimumab n = 7 (12%). Quantitative variables expressed in medians and ranges. Qualitative variables expressed in frequencies and percentages. Comparisons of means were computed using unpaired Student’s *t*-tests. Comparisons of proportions were computed with Chi-square tests. *p*-values in bold meaning statistically significant.

**Table 2:** Analyses of correlations of serum levels of follistatin and HAQ-DI with different characteristics of patients with RA.

	Follistatin		Disability Score	
Variable, n = 57	r	*p*-Value	r	*p*-Value
Age	0.088	0.515	0.009	0.947
Body mass index	0.126	0.349	0.165	0.220
Waist circumference	0.199	0.142	0.338	**0.011**
Waist-to-hip ratio	0.122	0.370	0.386	**0.003**
Skeletal muscle mass index (kg/m^2^)	0.077	0.569	0.032	0.814
Grip strength	−0.173	0.197	−0.546	**<0.001**
Gait speed	−0.124	0.357	−0.307	**0.020**
Physical performance	0.114	0.398	0.285	**0.032**
RA disease duration (years)	0.015	0.919	0.170	0.239
Tender joints	0.105	0.435	0.356	**0.007**
Swollen joints	0.217	0.105	0.302	**0.022**
HAQ-DI	0.491	**<0.001**	-------	-------
DAS28-ESR	0.344	**0.009**	0.471	**<0.001**
Erythrocyte sedimentation rate	0.398	**0.002**	0.318	**0.016**
Follistatin (pg/mL)	-------	-------	0.491	**<0.001**

Abbreviations: HAQ-DI: Health Assessment Questionnaire—Disability Index; DAS28-ESR: disease activity score of 28 joints. Disability score corresponds to the score obtained by HAQ-DI. Statistical tests: Pearson correlation of serum levels of follistatin and HAQ-DI score with clinical variables of RA. *p*-values in bold meaning statistically significant.

**Table 3:** Comparison of variables between subgroups of RA patients: (1) RA with functional disability compared to (2) RA with preserved physical function.

Variables	RA + Functional Disability (HAQ-DI ≥ 0.6) N = 25	RA + Preserved Physical Function (HAQ-DI < 0.6) N = 32	*p*-Value
Age, mean ± SD	57 ± 10	57 ± 8	0.867
Smoking, frequency (%)	9 (36)	8 (25)	0.368
Menopause, frequency (%)	18 (75)	25 (78)	0.784
Diabetes mellitus 2, frequency (%)	5 (20)	2 (6)	0.221
Hypertension, frequency (%)	11 (44)	9 (28)	0.213
Body mass index (kg/m^2^), mean ± SD	28.1 ± 5.6	26.8 ± 4.4	0.326
Waist circumference, mean ± SD	94.5 ± 12.8	87.1 ± 11.6	**0.029**
Waist-to-hip ratio, mean ± SD	0.92 ± 0.06	0.86 ± 0.08	**0.006**
Skeletal muscle mass index (kg/m^2^)	6.0 ± 1.0	6.2 ± 1.0	0.473
Grip strength, mean ± SD	7.9 ± 4.6	14.5 ± 5.1	**<0.001**
Decreased grip strength, frequency (%)	24 (96)	21 (66)	**0.005**
Gait speed, mean ± SD	0.77 ± 0.20	0.92 ± 0.20	**0.010**
Decreased gait speed, frequency (%)	20 (80)	19 (59)	0.096
Physical performance, mean ± SD	8.3 ± 2.4	6.9 ± 1.8	**0.014**
*RA Characteristics:*			
RA disease duration, mean ± SD	17.9 ± 12	15.2 ± 10	0.377
Tender joints count, mean ± SD	3.7 ± 4.6	1.9 ± 3.9	0.112
Tender joints count ≥ 4, frequency (%)	12 (48)	5 (16)	**0.008**
Swollen joints count, mean ± SD	2.2 ± 3.0	1.2 ± 2.6	0.180
Swollen joints count ≥ 4, frequency (%)	6 (24)	4 (13)	0.308
DAS28-ESR, mean ± SD	3.8 ± 1.5	2.8 ± 1.2	**0.008**
RA with active disease, frequency (%)	16 (64)	11 (34)	**0.026**
Erythrocyte sedimentation rate, mean ± SD	21.5 ± 6.9	18.4 ± 6.9	0.096
Follistatin (pg/mL), mean ± SD	218 ± 159	141 ± 59	**0.030**
Prednisone ≤ 10 mg/day, frequency (%)	16 (64)	11 (35)	**0.025**
Methotrexate, frequency (%)	22 (88)	30 (94)	0.645
Sulfasalazine, frequency (%)	18 (72)	23 (72)	0.992
Leflunomide, frequency (%)	13 (52)	16 (50)	0.881
Chloroquine, frequency (%)	17 (68)	18 (56)	0.366
Anti-TNF agents, frequency (%)	7 (28)	12 (38)	0.450

Decreased grip strength: ≤16 kg; decreased gait speed: <1.0 m/s; HAQ-DI: Health Assessment Questionnaire—Disability Index; impaired functioning (HAQ-DI ≥ 0.6); DAS28-ESR: disease activity score of 28 joints; RA with active disease: DAS28-ESR ≥ 2.6; ESR: erythrocyte sedimentation rate; RA disease activity: DAS28-ESR ≥ 2.6; tender or swollen joints count ≥ 4; prednisone: <10 mg/day. Means were compared with Student’s *t*-test. Proportions were compared with Chi-square tests (Fisher exact tests when appropriate). *p*-values in bold meaning statistically significant.

**Table 4:** Subanalysis comparing follistatin concentrations between users versus non-users of different drugs.

Drug	n (%)	Follistatin Levels, Mean ± SD	*p*-Value
Methotrexate users	52 (92)	181.2 ± 122.8	0.19
Non-methotrexate users	5 (8)	107.8 ± 30.3	
Sulfasalazine users	41 (72)	182.0 ± 125.0	0.47
Non-sulfasalazine users	16 (28)	156.3 ± 104.9	
Leflunomide users	29 (51)	177.8 ± 108.0	0.85
Non-leflunomide users	28 (49)	171.6 ± 132.0	
Chloroquine users	35 (61)	190.2 ± 130.7	0.22
Non-chloroquine users	22 (39)	150.3 ± 96.5	
Anti-TNF users	19 (33)	194.6 ± 155.9	0.38
Non-anti-TNF users	38 (67)	164.9 ± 97.1	
Prednisone users	27 (47)	180.9 ± 107.7	0.72
Non-prednisone users	30 (53)	169.3 ± 130.5	

Comparisons of means of drugs were computed using unpaired Student’s *t*-tests.

## Data Availability

Data used for supporting the results of this work are available upon request to the author Laura Gonzalez-Lopez: ldelcarmen.gonzalez@academicos.udg.mx, or lauraacademicoudg@gmail.com.

## Abbreviations

Abbreviations used in this manuscript:

ACR	American College of Rheumatology
ActRIIA/ActRIIB	Activin Receptor Type IIA/B
ALT	Alanine Aminotransferase
AST	Aspartate Aminotransferase
BMI	Body Mass Index
CKD	Chronic Kidney Disease
DXA	Dual-Energy X-ray Absorptiometry
DMARDs	Disease-Modifying Anti-Rheumatic Drugs
DAS28-ESR	Disease Activity Score of 28 Joints—Erythrocyte Sedimentation Rate
ELISA	Enzyme-Linked Immunosorbent Assay
ESR	Erythrocyte Sedimentation Rate
HAQ-DI	Health Assessment Questionnaire-Disability Index
IL-6	Interleukin 6
IL-β	Interleukin 1β
IMAT	Intermuscular Adipose Tissue
INTEC	Instituto de Terapeutica Experimental y Clinica
MyHC	Myosin Heavy-Chain
RA	Rheumatoid Arthritis
SMMI	Skeletal Muscle Mass Index
TGFβ	Transforming Growth Factor Beta
TNF-α	Tumor Necrosis Factor-α

## References

[1] Aletaha D., Neogi T., Silman A.J., Funovits J., Felson D.T., Bingham C.O., Birnbaum N.S., Burmester G.R., Bykerk V.P., Cohen M.D. (2010). Corretion in 2010 Rheumatoid Arthritis Classification Criteria: An American College of Rheumatology/European League Against Rheumatism Collaborative Initiative. Ann. Rheum. Dis..

[2] Smolen J.S., Aletaha D., McInnes I.B. (2016). Rheumatoid Arthritis. Lancet.

[3] Song Y., Chen Y., Wen L., He B., Ding Y., Liu M., Tang F., Wang L., Wu J., Deng X. (2024). Health-Related Quality of Life Profiles in Patients with Rheumatoid Arthritis: A Latent Profile Analysis. Front. Public Health.

[4] Santo R.C.D.E., Baker J.F., Dos Santos L.P., Silva J.M.S., Filippin L.I., Portes J.K.S., Brenol C.V., Chakr R.M.D.S., Xavier R.M. (2023). Changes in Physical Function over Time in Rheumatoid Arthritis Patients: A Cohort Study. PLoS ONE.

[5] Cardiel M.H., Abello-Banfi M., Ruiz-Mercado R., Alarcon-Segovia D. (1993). How to Measure Health Status in Rheumatoid Arthritis in Non-English Speaking Patients: Validation of a Spanish Version of the Health Assessment Questionnaire Disability Index (Spanish HAQ-DI). Clin. Exp. Rheumatol..

[6] Teuwen M.M.H., van Wissen M.A.T., Peter W.F., van Schaardenburg D., van den Ende C.H.M., Gademan M.G.J., van Weely S.F.E. (2024). The Extent and Nature of Functional Limitations According to the Health Assessment Questionnaire Disability Index in Patients with Rheumatoid Arthritis and Severe Functional Disability. J. Clin. Med..

[7] Coppers B., Heinrich S., Tascilar K., Phutane U., Kleyer A., Simon D., Bräunig J., Penner J., Vossiek M., Schönau V. (2025). Sensor-Assessed Grasping Time as a Biomarker of Functional Impairment in Rheumatoid Arthritis. Sci. Rep..

[8] Jin H., Wang G., Lu Q., Rawlins J., Chen J., Kashyap S., Charlesworth O., Xu D., Dai L., Zhu S. (2025). Pathophysiology of Myopenia in Rheumatoid Arthritis. Bone Res..

[9] Baig M.H., Ahmad K., Moon J.S., Park S.-Y., Ho Lim J., Chun H.J., Qadri A.F., Hwang Y.C., Jan A.T., Ahmad S.S. (2022). Myostatin and Its Regulation: A Comprehensive Review of Myostatin Inhibiting Strategies. Front. Physiol..

[10] Nguyen H.Q., Iskenderian A., Ehmann D., Jasper P., Zhang Z., Rong H., Welty D., Narayanan R. (2020). Leveraging Quantitative Systems Pharmacology Approach into Development of Human Recombinant Follistatin Fusion Protein for Duchenne Muscular Dystrophy. CPT Pharmacomet. Syst. Pharmacol..

[11] Wang S., Fang L., Cong L., Chung J.P.W., Li T.C., Chan D.Y.L. (2022). Myostatin: A Multifunctional Role in Human Female Reproduction and Fertility—A Short Review. Reprod. Biol. Endocrinol..

[12] Wu C., Borné Y., Gao R., López Rodriguez M., Roell W.C., Wilson J.M., Regmi A., Luan C., Aly D.M., Peter A. (2021). Elevated Circulating Follistatin Associates with an Increased Risk of Type 2 Diabetes. Nat. Commun..

[13] Stefan N., Schick F., Birkenfeld A.L., Häring H.-U., White M.F. (2023). The Role of Hepatokines in NAFLD. Cell Metab..

[14] Correa-de-Araujo R., Addison O., Miljkovic I., Goodpaster B.H., Bergman B.C., Clark R.V., Elena J.W., Esser K.A., Ferrucci L., Harris-Love M.O. (2020). Myosteatosis in the Context of Skeletal Muscle Function Deficit: An Interdisciplinary Workshop at the National Institute on Aging. Front. Physiol..

[15] Chang K.-V., Wu W.-T., Chen Y.-H., Chen L.-R., Hsu W.-H., Lin Y.-L., Han D.-S. (2023). Enhanced Serum Levels of Tumor Necrosis Factor-α, Interleukin-1β, and -6 in Sarcopenia: Alleviation through Exercise and Nutrition Intervention. Aging (Albany NY).

[16] Dondero K., Friedman B., Rekant J., Landers-Ramos R., Addison O. (2024). The Effects of Myosteatosis on Skeletal Muscle Function in Older Adults. Physiol. Rep..

[17] Andonian B.J., Huffman K.M. (2020). Skeletal Muscle Disease in Rheumatoid Arthritis: The Center of Cardiometabolic Comorbidities?. Curr. Opin. Rheumatol..

[18] Du Y., Xu C., Shi H., Jiang X., Tang W., Wu X., Chen M., Li H., Zhang X., Cheng Q. (2021). Serum Concentrations of Oxytocin, DHEA and Follistatin Are Associated with Osteoporosis or Sarcopenia in Community-Dwelling Postmenopausal Women. BMC Geriatr..

[19] Fife E., Kostka J., Kroc Ł., Guligowska A., Pigłowska M., Sołtysik B., Kaufman-Szymczyk A., Fabianowska-Majewska K., Kostka T. (2018). Relationship of Muscle Function to Circulating Myostatin, Follistatin and GDF11 in Older Women and Men. BMC Geriatr..

[20] Liaw F.-Y., Kao T.-W., Fang W.-H., Han D.-S., Chi Y.-C., Yang W.-S. (2016). Increased Follistatin Associated with Decreased Gait Speed among Old Adults. Eur. J. Clin. Investig..

[21] Pazokian F., Amani-Shalamzari S., Rajabi H. (2022). Corretion in Effects of Functional Training with Blood Occlusion on the Irisin, Follistatin, and Myostatin Myokines in Elderly Men. Eur. Rev. Aging Phys. Act..

[22] Miyamoto T., Carrero J.J., Qureshi A.R., Anderstam B., Heimbürger O., Bárány P., Lindholm B., Stenvinkel P. (2011). Circulating Follistatin in Patients with Chronic Kidney Disease: Implications for Muscle Strength, Bone Mineral Density, Inflammation, and Survival. Clin. J. Am. Soc. Nephrol..

[23] Echeverria I., Besga A., Sanz B., Amasene M., Hervás G., Barroso J., Rodriguez-Larrad A., Irazusta J. (2021). Identification of Frailty and Sarcopenia in Hospitalised Older People. Eur. J. Clin. Investig..

[24] Kurose S., Onishi K., Miyauchi T., Takahashi K., Kimura Y. (2023). Serum Follistatin Levels Are Independently Associated with Exercise Tolerance in Patients with Obesity. Endocr. Res..

[25] Kerschan-Schindl K., Ebenbichler G., Föeger-Samwald U., Leiss H., Gesslbauer C., Herceg M., Stummvoll G., Marculescu R., Crevenna R., Pietschmann P. (2019). Rheumatoid Arthritis in Remission: Decreased Myostatin and Increased Serum Levels of Periostin. Wien. Klin. Wochenschr..

[26] van Groen M.M., ten Klooster P.M., Taal E., van de Laar M.A.F.J., Glas C.A.W. (2010). Application of the Health Assessment Questionnaire Disability Index to Various Rheumatic Diseases. Qual. Life Res..

[27] de Sordi C.M., Dos Reis-Neto E.T., Keppeke G.D., Shinjo S.K., Sato E.I. (2022). Serum Myostatin and Follistatin Levels in Patients with Dermatomyositis and Polymyositis. J. Clin. Rheumatol..

[28] Samaan S.F., Taha S.I. (2022). The Impact of Metabolic Syndrome on Quality of Life Among Individuals with Knee Osteoarthritis Living in Egypt. Clin. Med. Insights Arthritis Musculoskelet. Disord..

[29] Son K.M., Kang S.H., Seo Y.I., Kim H.A. (2021). Association of Body Composition with Disease Activity and Disability in Rheumatoid Arthritis. Korean J. Intern. Med..

[30] Melikoğlu M.A. (2017). Presarcopenia and Its Impact on Disability in Female Patients with Rheumatoid Arthritis. Arch. Rheumatol..

[31] Lopes A.J., Justo A.C., Ferreira A.S., Guimaraes F.S. (2017). Systemic Sclerosis: Association between Physical Function, Handgrip Strength and Pulmonary Function. J. Bodyw. Mov. Ther..

[32] Özsoy Z., Hafızoğlu M., Öztürk Z., Şahiner Z., Karaduman D., Uzun G.S., Ünaldı E., Tahıllıoğlu Y., Halil M.G., Özsoy Z. (2024). Improvement in Rheumatoid Sarcopenia with Biological Therapy; Muscle Ultrasound Study. J. Turk. Soc. Rheumatol..

[33] Hammer H.B., Jensen Hansen I.M., Järvinen P., Leirisalo-Repo M., Ziegelasch M., Agular B., Terslev L. (2021). Rheumatoid Arthritis Patients with Predominantly Tender Joints Rarely Achieve Clinical Remission despite Being in Ultrasound Remission. Rheumatol. Adv. Pr..

[34] Feng J., Yu L., Fang Y., Zhang X., Li S., Dou L. (2024). Correlation between Disease Activity and Patient-Reported Health-Related Quality of Life in Rheumatoid Arthritis: A Cross-Sectional Study. BMJ Open.

[35] Hulmi J.J., Nissinen T.A., Penna F., Bonetto A. (2021). Targeting the Activin Receptor Signaling to Counteract the Multi-Systemic Complications of Cancer and Its Treatments. Cells.

[36] Sartori R., Romanello V., Sandri M. (2021). Mechanisms of Muscle Atrophy and Hypertrophy: Implications in Health and Disease. Nat. Commun..

[37] Iyer C.C., Chugh D., Bobbili P.J., Blatnik A.J., Crum A.E., Yi A.F., Kaspar B.K., Meyer K.C., Burghes A.H.M., Arnold W.D. (2021). Follistatin-Induced Muscle Hypertrophy in Aged Mice Improves Neuromuscular Junction Innervation and Function. Neurobiol. Aging.

[38] Kramerova I., Marinov M., Owens J., Lee S.-J., Becerra D., Spencer M.J. (2020). Myostatin Inhibition Promotes Fast Fibre Hypertrophy but Causes Loss of AMP-Activated Protein Kinase Signalling and Poor Exercise Tolerance in a Model of Limb-Girdle Muscular Dystrophy R1/2A. J. Physiol..

[39] Rybalka E., Timpani C.A., Debruin D.A., Bagaric R.M., Campelj D.G., Hayes A. (2020). The Failed Clinical Story of Myostatin Inhibitors against Duchenne Muscular Dystrophy: Exploring the Biology behind the Battle. Cells.

[40] Agrawal S., Chakole S., Shetty N., Prasad R., Lohakare T., Wanjari M. (2023). Exploring the Role of Oxidative Stress in Skeletal Muscle Atrophy: Mechanisms and Implications. Cureus.

[41] Escalante A., Haas R.W., del Rincón I. (2004). Measurement of Global Functional Performance in Patients with Rheumatoid Arthritis Using Rheumatology Function Tests. Arthritis Res. Ther..

[42] Thyberg I., Hass U.A.M., Nordenskiöld U., Gerdle B., Skogh T. (2005). Activity Limitation in Rheumatoid Arthritis Correlates with Reduced Grip Force Regardless of Sex: The Swedish TIRA Project. Arthritis Rheum..

[43] Meng C.F., Lee Y., Schieir O., Valois M.-F., Butler M., Boire G., Hazlewood G., Hitchon C., Keystone E., Tin D. (2024). Having More Tender Than Swollen Joints Is Associated with Worse Function and Work Impairment in Patients with Early Rheumatoid Arthritis. ACR Open Rheumatol..

[44] Krause M.L., Crowson C.S., Bongartz T., Matteson E.L., Michet C.J., Mason T.G., Persellin S.T., Gabriel S.E., Davis J.M. (2015). Determinants of Disability in Rheumatoid Arthritis: A Community-Based Cohort Study. Open Rheumatol. J..

[45] Pfeiffer B.M., Krenzer S., Dockhorn R., Schwenke R., Schwenke H., Waehrisch J., Kraus E. (2013). Impact of Modified-Release Prednisone on Functional Ability in Patients with Rheumatoid Arthritis. Rheumatol. Int..

[46] Maska L., Anderson J., Michaud K. (2011). Measures of Functional Status and Quality of Life in Rheumatoid Arthritis: Health Assessment Questionnaire Disability Index (HAQ), Modified Health Assessment Questionnaire (MHAQ), Multidimensional Health Assessment Questionnaire (MDHAQ), Health Assessment Questionnaire II (HAQ-II), Improved Health Assessment Questionnaire (Improved HAQ), and Rheumatoid Arthritis Quality of Life (RAQoL). Arthritis Care Res. (Hoboken).

[47] Prevoo M.L., van ’t Hof M.A., Kuper H.H., van Leeuwen M.A., van de Putte L.B., van Riel P.L. (1995). Modified Disease Activity Scores That Include Twenty-Eight-Joint Counts. Development and Validation in a Prospective Longitudinal Study of Patients with Rheumatoid Arthritis. Arthritis Rheum..

[48] Fransen J., van Riel P.L.C.M. (2005). The Disease Activity Score and the EULAR Response Criteria. Clin. Exp. Rheumatol..

[49] Dougados M., Brault Y., Logeart I., van der Heijde D., Gossec L., Kvien T. (2012). Defining Cut-off Values for Disease Activity States and Improvement Scores for Patient-Reported Outcomes: The Example of the Rheumatoid Arthritis Impact of Disease (RAID). Arthritis Res. Ther..

[50] Garrow J.S. (1988). Obesity and Related Diseases.

[51] Megnien J.L., Denarie N., Cocaul M., Simon A., Levenson J. (1999). Predictive Value of Waist-to-Hip Ratio on Cardiovascular Risk Events. Int. J. Obes. Relat. Metab. Disord..

[52] Lewiecki E.M., Binkley N., Morgan S.L., Shuhart C.R., Camargos B.M., Carey J.J., Gordon C.M., Jankowski L.G., Lee J.-K., Leslie W.D. (2016). Best Practices for Dual-Energy X-Ray Absorptiometry Measurement and Reporting: International Society for Clinical Densitometry Guidance. J. Clin. Densitom..

[53] Cawthon P.M. (2015). Assessment of Lean Mass and Physical Performance in Sarcopenia. J. Clin. Densitom..

[54] Yamada Y., Yamada M., Yoshida T., Miyachi M., Arai H. (2021). Validating Muscle Mass Cutoffs of Four International Sarcopenia-working Groups in Japanese People Using DXA and BIA. J. Cachexia Sarcopenia Muscle.

[55] Spijkerman D.C., Snijders C.J., Stijnen T., Lankhorst G.J. (1991). Standardization of Grip Strength Measurements. Effects on Repeatability and Peak Force. Scand. J. Rehabil. Med..

[56] Alley D.E., Shardell M.D., Peters K.W., McLean R.R., Dam T.-T.L., Kenny A.M., Fragala M.S., Harris T.B., Kiel D.P., Guralnik J.M. (2014). Grip Strength Cutpoints for the Identification of Clinically Relevant Weakness. J. Gerontol. A Biol. Sci. Med. Sci..

[57] Boshnjaku A., Krasniqi E. (2024). Diagnosing Sarcopenia in Clinical Practice: International Guidelines vs. Population-Specific Cutoff Criteria. Front. Med..

